# Dual HER2 blockade: preclinical and clinical data

**DOI:** 10.1186/s13058-014-0419-5

**Published:** 2014-07-31

**Authors:** Tejal A Patel, Bhuvanesh Dave, Angel A Rodriguez, Jenny C Chang, Edith A Perez, Gerardo Colon-Otero

**Affiliations:** 1 Houston Methodist Cancer Center, 6445 Main Street, P21-34 Houston, 77030 TX USA; 2 Department of Medicine, Weill Cornell Medical College, 1300 York Avenue, New York, 10065 NY USA; 3 Division of Hemotology and Oncology, College of Medicine, Mayo Clinic, 4500 San Pablo Rd S., Jacksonville, 32224 FL USA

## Abstract

The estrogen receptor and human epidermal growth factor receptor (HER) signaling pathways are the dominant drivers of cell proliferation and survival in the majority of human breast cancers. Not surprisingly, targeting these pathways provides the most effective therapies in appropriately selected patients. However, *de novo* and acquired resistance remain major obstacles to successful treatment. By increasing the understanding of the molecular mechanisms of combined HER2-targeted therapies, we aim to be better able to select patients who would respond to these treatments and understand some of the mechanisms of resistance to HER2-targeted treatments. Recent studies have demonstrated an increased effectiveness of dual targeted HER2 therapies against HER2-amplified breast cancer as compared with single blockade. These studies have resulted in the recent US Food and Drug Administration approval of the combination of taxane chemotherapy with pertuzumab and trastuzumab in the first-line metastatic setting as well as an accelerated approval in the neoadjuvant setting. Another mechanism for overcoming resistance to HER2 targeted therapies is the antibody-drug conjugate trastuzumab-emtansine, which targets the HER2 receptor conjugated to the potent antimicrotubule agent mertansine, allowing for intracellular release of the cytotoxic drug. Studies evaluating the efficacy of dual blockade with antibody-drug conjugate are currently ongoing. This article reviews recent data on different combinations of anti-HER2 treatments as well as ongoing and future research in this area.

**Electronic supplementary material:**

The online version of this article (doi:10.1186/s13058-014-0419-5) contains supplementary material, which is available to authorized users.

## Introduction

Recognition of the impact of human epidermal growth factor receptor (HER)-2 overexpression or amplification in approximately 15 to 20% of all cases of invasive breast cancer has resulted in the development of multiple drugs that inhibit the proliferative signal pathway associated with this molecular alteration. The incorporation of HER2-directed therapy has improved the overall survival (OS) of metastatic breast cancer (MBC) patients by greater than 20% and has increased the cure rate of breast cancer in the adjuvant setting by approximately 30 to 40% [1],[2]. Despite this, approximately 5,000 patients with HER2-overexpressing breast cancer die each year in the USA [3].

The HER family of transmembrane type I receptor tyrosine kinases includes four receptors (HER1 to HER4) that play an important role in cell processes including cell proliferation and survival. HER2 does not require ligand activation and can form homodimers or can interact with the other HER family receptors by forming heterodimers that lead to the activation of the HER2 tyrosine kinase. HER3 has only a weak intrinsic tyrosine kinase activity that activates HER2 by forming heterodimers with HER2, leading to the strongest preclinical mitogenic signals of all possible HER receptor dimer combinations [4]. Upon ligand binding to the active domain of HER1, HER3 or HER4, these receptors can activate homodimeric or heterodimeric receptor complexes - but they preferentially recruit HER2 into a heterodimeric complex in which the HER2 kinase can modulate receptor internalization and prolong signal transduction. Conformational changes occur upon dimerization, leading to autophosphorylation and initiation of divergent signal transduction cascades [5]. These signaling pathways from these receptor heterodimers are not absolutely linear and some of their functions may overlap; laboratory data generally indicate that HER1/HER2 heterodimers activate cell proliferation by the extracellular signal-regulated kinase 1/2-mitogen-activated protein kinase pathway [6], while HER2/HER3 heterodimers predominantly activate the phosphoinositide-3-kinase (PI3K)/AKT cell survival pathway [7].

## Approved HER2-targeted drugs for the treatment of HER2-positive breast cancer

Several drugs have been developed and are in clinical use to block the HER pathway, most aimed at the receptor level.

Trastuzumab, a monoclonal antibody directed against HER2, became the first HER2-directed therapy for MBC and the first monoclonal antibody against cancer approved by the US Food and Drug Administration (FDA) in 1998 [2]. Trastuzumab has been theorized to induce cell death in HER2-overexpressing breast cancer cells by multiple mechanisms including antibody-dependent cell-mediated cytotoxicity, induction of apoptosis and inactivation of HER2-mediated cell proliferation signaling [3]. A phase III clinical trial showed the effectiveness of trastuzumab in synergizing with chemotherapy by increasing the response rate and improving the OS of patients with MBC when compared with chemotherapy alone [2]. Trastuzumab is also commonly used in the refractory metastatic setting in combination with a wide range of chemotherapy agents. Use of trastuzumab is also pivotal to patient management in the adjuvant setting, as it improves disease-free survival (DFS) and OS when added to chemotherapy [1].

Lapatinib is an orally active dual HER1/HER2 kinase inhibitor that blocks signal transduction pathways. Lapatinib reduces tyrosine phosphorylation of HER1 and HER2, as well as activation of extracellular signal-regulated kinase 1/2-mitogen-activated protein kinase and PI3K/AKT, affecting downstream effectors of both proliferation and survival [8]. Lapatinib has demonstrated activity in patients with HER2-overexpressing MBC after escape from trastuzumab and is currently approved as second-line therapy for MBC patients after trastuzumab failure [9],[10]. However, comparative phase III trials of chemotherapy with either trastuzumab or lapatinib suggested that trastuzumab was the optimal anti-HER2 therapy to select in this first-line MBC setting.

Pertuzumab, a recombinant humanized monoclonal antibody (2C4), binds to extracellular domain II of the HER2 receptor and blocks its ability to dimerize with other HER receptors, in particular HER2-HER3 complexes [11]. Pertuzumab was approved in combination with chemotherapy and trastuzumab for the first-line treatment of HER2-positive MBC and for the neoadjuvant therapy of HER2-positive breast cancer, based on data demonstrating improvement in progression-free survival and OS as compared with trastuzumab-based chemotherapy in patients with MBC and a higher rate of pathological complete remissions in the neoadjuvant setting [12].

In addition to these receptor targeted therapies, a new class of antibody-drug conjugate (ADC) has recently shown superior clinical activity. Ado-trastuzumab-emtansine (T-DM1) is an ADC that incorporates the HER2-targeted antitumor properties of trastuzumab with the cytotoxic activity of the microtubule-inhibitory agent mertansine (derivative of maytansine); the antibody and the cytotoxic agent are conjugated by means of a unique stable linker. T-DM1 allows intracellular drug delivery specifically to HER2-overexpressing cells, thereby improving the therapeutic index and minimizing exposure of normal tissue. T-DM1 has demonstrated survival and superior tolerability over the lapatinib/capecitabine combination in the refractory HER2-positive advanced setting, and has received regulatory agency approval for such situations [13],[14].

Table 1 presents the pivotal trials leading to US FDA approval of the different drugs targeting HER2 and the different approved combinations. This approval has converted a disease that had the worst prognosis of all breast cancer subtypes into one with the best prognosis. Of interest is the fact that only a minority of breast cancer cases (20%) benefit from these treatments, which implies that these treatments may not have been proven to be of benefit if they had been applied to all breast cancer patients instead of being limited to the HER2-amplified subset, illustrating the importance of appropriate patient subset selection based on the biology of the tumor for successful drug discovery and development.

**Table 1:** **Chronological summary of US Food and Drug Administration approved anti-HER2 treatments in HER2-amplified breast cancer**

Year of approval	Cancer stage	Agent	Study name	Chemotherapy
1998 [2]	First-line metastatic	T	-	Pac or AC
1998 [2]	Second-line and third-line metastatic	T	-	None
2006 [1],[15]	Adjuvant	T	B31/N9831/BCIG006	AC → Pac, TCH
2006 [16]	Metastatic	L	-	Capecitabine
2009 [17]	Metastatic	L + AI	TAnDEM	None
2012 [18]	Metastatic	P + T	CLEOPATRA	Docetaxel
2013 [13]	Metastatic	TDM-1	EMILIA	None
2013 [19]	Neoadjuvant	P + T	NeoSphere	FEC → Pac, TCH

AI, aromatase inhibitor; AC, doxorubicin and cyclophosphamide; FEC, fluorouracil, epirubicin, cyclophosphamide, HER, human epidermal growth factor receptor; L, lapatinib; P, pertuzumab; Pac, paclitaxel; T, trastuzumab; TCH, docetaxel, carboplatin and trastuzumab; TDM-1, ado-trastuzumab emtansine.

Despite the success of these agents that target the HER family as single agents, there are a number of escape mechanisms from HER-targeted therapies. Clinically, a more complete blockade of the HER receptor layer has been shown to be therapeutically meaningful in prolonging survival in patients. With incomplete blockade of the receptor input layer, proliferative and survival signals can be generated from several different dimer pairs. The idea that the redundancy in the input layer of the network might provide an escape mechanism around a single-agent block has been explored in preclinical trials and neoadjuvant trials as well as in adjuvant trials. Dual HER2 blockade is defined as a more complete blockade of the HER2 and HER signaling pathway by combining two inhibitors with complementary mechanisms of action. In this article, we will review the data supporting these findings and the plans for further evaluation of dual HER2 blockade.

## Why is dual HER2 blockade more effective than single-drug blockade: causes of resistance to single-agent HER2 blockade

Despite the success in MBC, responses to single-agent trastuzumab are limited and cancer will eventually progress. Many patients treated with adjuvant trastuzumab will be cured of the disease, but disease will recur in some of them. This suggests that both *de novo* and acquired mechanisms of drug resistance exist. Several possible causes of resistance to both trastuzumab and lapatinib have been identified in preclinical studies. Few of these have been prospectively validated in clinical trials. There is enough indication to suggest that some of them do limit the effectiveness of HER2-directed therapy, particularly when these agents are used as single agents.

Some of the proposed mechanisms for resistance to trastuzumab include incomplete blockade of heterodimeric signaling or increased signaling through alternative signal transduction pathways, including upregulation of ligands or the receptors themselves [20], constitutive activation of the PI3K/Akt pathway due to loss of phosphatase and tensin homolog (PTEN) or activating mutations of PI3KCA or amplification of cyclin E [21],[22], increased transforming growth factor-alpha expression, and the presence of altered forms of HER2 [23],[24], which inhibits HER2-trastuzumab interactions. Escape pathways such as estrogen receptor (ER) or insulin-like growth factor receptor signaling have also been implicated in resistance. The data regarding PTEN loss and PI3K mutation and trastuzumab have been conflicting. Preclinical and smaller clinical studies have suggested PTEN loss associated with resistance to trastuzumab [25], although a larger study (*n* = 1,082) did not find an impact on DFS [26].

Mechanisms for lapatinib resistance are less well established and are hypothesized to include increased expression of AXL, a membrane-bound receptor tyrosine kinase with transforming ability [27], enhanced ER signaling through transcription factor FOXO3a [28], and upregulation of HER3 transcription [20]. There are discordant data with regards to PTEN loss and PI3K mutation and use of lapatinib in preclinical and small clinical studies [25],[29].

The existence of resistance to trastuzumab and the development of resistance after exposure to trastuzumab are the main reasons for recurrences. Enhanced blockade of HER2 signaling with dual HER2 therapies may result in decreased recurrences and ultimately improve survival.

## Evidence for improved activity of combination anti-HERtherapy

Numerous recent and ongoing multicenter studies have focused on the benefits and toxicity of adding single-agent or dual HER2 targeting to chemotherapy. In the neoadjuvant setting, almost all of the trials used a chemotherapy backbone (taxane only or anthracycline/taxane or taxane/platinum), with the exception of TBCRC 006 and a subset of patients treated on NeoSphere that only included the targeted therapies without chemotherapy. Using combinations of inhibitors (for example, trastuzumab with pertuzumab or lapatinib with trastuzumab), higher responses with higher pathologic complete responses (pCRs) have been observed (Tables 2 and 3).

**Table 2:** **Published or presented multicenter neoadjuvant trials in HER2-positive disease including lapatinib**

Study	Neoadjuvant regimen	pCR breast and lymph node T (%)	pCR breast and lymph node L (%)	pCR breast and lymph node combination T + L (%)	pCR breast and lymph node combination T + L without chemotherapy (%)	Compliance	Statistical significance
NeoALTTO [12],[34]	6 weeks T and/or L → WP × 12 weeks plus T and/or L (*n* = 455)	27.6	20	46.8	NA	93% T, 66% L, 61% T + L	Between T + L and T, *P* = 0.0007
CHER-LOB [35]	WP × 12 weeks → FEC × 4 every 3 weeks plus T and/or L throughout (*n* = 121)	25	26.3	46.7	NA	NR T, 69% L, 83% T + L	No comparisons among treatment regimens planned. Exploratory analysis between T + L vs. T, risk ratio = 1.81, *P* = 0.019
NSABP 41 [36]	AC × 4 → WP × 12 weeks plus T and/or L (*n* = 519)	49.4	47.7	60.2	NA	77% T, 65% L, 63% T + L	Between T + L vs. T, *P* = 0.056; between L vs. T, *P* = 0.78; not significant
CALGB 40601 [37]	WP × 16 weeks plus T and/or L (*n* = 299)	43	29	52	NA	93% T, 66% L, 87% T + L	*P* = 0.11; not significant
TRIO US B07 [38]	T and/or L × 21 days → TCH × 6 cycles plus T and/or L throughout (*n* = 130)	47	25	52	NA	100% T, 72% L, 73% T + L	Between T + L vs. L, *P* = 0.02; between T and L, *P* = 0.07
TBCRC 006 [32]	T and L × 12 weeks; ER + letrozole ± G (*n* = 66)	NA	NA	NA	22	NR	Single-arm study
LPT 109096 [39]	2 weeks T and/or L → FEC 75 × 4 every 3 weeks → WP × 12 weeks + T and/or L throughout (*n* = 100)	54	45	74	NA	NR	Not powered to compare responses between arms

AC, adriamycin, cyclophosphamide; ER, estrogen receptor; FEC, fluorouracil, epirubicin, cyclophosphamide; G, goserelin; HER, human epidermal growth factor receptor; L, lapatinib; NA, not applicable; NR, not reported; pCR, pathologic complete response; T, trastuzumab; TCH, docetaxel, carboplatin and trastuzumab; WP, weekly paclitaxel.

**Table 3:** **Published or presented multicenter neoadjuvant trials in HER2-positive disease including pertuzumab**

Study	Neoadjuvant regimen	pCR breast and lymph node T (%)	pCR breast and lymph node P (%)	pCR breast and lymph node combination T + P (%)	pCR breast and lymph node combination T + P without chemotherapy (%)	Statistical significance
NeoSphere [19]	D x 4 with T and/or P	21.5	17.7	39.3	11.2	NR for pCR breast and lymph node. For pCR breast only between T + P and T, *P* = 0.0141
TRYPHAENA [43]	Arm A, FECPH → DPH; Arm B, FEC → DPH; Arm C, TCHP	50.7	45.3	51.9	NA	Primary endpoint was cardiac safety, *P* value NR

D, docetaxel; DPH, docetaxel, pertuzumab and trastuzumab; FEC, fluorouracil, epirubicin, cyclophosphamide; FECPH, fluorouracil, epirubicin, cyclophosphamide, pertuzumab and trastuzumab; HER, human epidermal growth factor receptor; NA, not applicable; NR, not reported; P, pertuzumab; pCR, pathologic complete response; T, trastuzumab; TCHP, docetaxel, carboplatin, trastuzumab and pertuzumab.

### Lapatinib and trastuzumab

#### Preclinical MCF-7/HER2 mouse xenograft studies

Trastuzumab, pertuzumab, lapatinib and gefitinib represent a group of therapeutic agents that target the HER family by different molecular mechanisms. These drugs, when used as single agents in the MCF7/HER2-18 xenograft model, restored or enhanced sensitivity to tamoxifen. However, tumor growth inhibition lasted only 2 to 3 months before resistance to treatments occurred and tumor growth resumed. Preclinical studies in animal models evaluating the efficacy of various drug combinations have shown that the combination HER-targeting therapy with estrogen deprivation more effectively induced apoptosis, reduced levels of p-AKT and mitogen-activated protein kinase, inhibited proliferation and was capable of eradicating HER2 overexpressing xenografts in mice [30],[31]. Based on these results, neoadjuvant studies with trastuzumab and lapatinib together with estrogen deprivation were designed (TBCRC 006, see below) [32].

#### Clinical studies

In the metastatic setting, a phase III clinical trial comparing lapatinib versus lapatinib and trastuzumab in 296 patients who had progressed on a trastuzumab-containing regimen demonstrated an improvement in progression-free survival (hazard ratio (HR), 0.73; 95% confidence interval, 0.57 to 0.93; *P* = 0.08) and a trend towards improved OS (HR, 0.75; 95% confidence interval, 0.53 to 1.07; *P* = 0.106) in patients receiving the combination [33]. This observation provided further evidence for combined HER2 blockade as well as continued use of trastuzumab beyond disease progression.

Six randomized neoadjuvant trials and one nonrandomized neoadjuvant trial as well as one adjuvant trial have evaluated the role of dual targeted therapies with lapatinib and trastuzumab (Table 2). Two large studies were conducted with a taxane-only backbone with anthracycline chemotherapy given after surgery. In the phase III study of NeoALTTO, 455 patients received paclitaxel with lapatinib, trastuzumab or the combination [12]. The dual-therapy arm had significantly improved pCR in the breast and axilla compared with trastuzumab alone (46.8% vs. 27.6%, *P* = 0.0007) [12]. The pCR rates were higher in patients with hormone receptor-negative tumors than those of hormone receptor-positive tumors in all groups (61.3 vs. 41.6%). A recent update of the study demonstrated that patients who achieved pCR had significantly better event-free survival (EFS) (86% vs. 72%; HR, 0.38; *P* = 0.003) and OS (94% vs. 87%; HR 0.35; *P* = 0.005) compared with no pCR [34]. These results did not address the question of whether the higher pCR rate achieved with dual therapy translated into better EFS or OS because the NeoALTTO trial was underpowered to detect moderate differences in EFS and OS.

The adjuvant study ALTTO did not meet the primary endpoint of improved DFS with the addition of lapatinib to trastuzumab compared with trastuzumab as adjuvant treatment for HER2-positive early breast cancer [40]. ALTTO has raised questions regarding the use of an increase in pCR rate as a surrogate endpoint for improved DFS in the adjuvant setting and the effect of dual blockade on long-term outcome. ALTTO demonstrates that a large proportion of patients with HER2-positive early breast cancer do not derive benefit with dual blockade of lapatinib and trastuzumab. Whether a benefit exists in a higher risk population or in a subset of patients identified by a biomarker or a different dual blockade should be further evaluated. APHINITY is an adjuvant trial evaluating dual blockade of pertuzumab and trastuzumab versus trastuzumab in a high-risk population (node-positive) and will further answer the question regarding the benefit of dual blockade.

The CALGB 40601 trial randomized 305 patients to receive weekly paclitaxel for 16 weeks with trastuzumab alone or with the combination trastuzumab and lapatinib [37]. The pCR rate in the breast and axilla was numerically higher at 52% with the combination compared with 43% for the chemotherapy and trastuzumab arm, but was not statistically significant. This could have been due to underlying differences in the study population and highlights the need to identify tumors that can benefit with dual versus single HER2 targeting, possibly looking at the hormone receptor differences.

Three studies have evaluated the combination of lapatinib and trastuzumab with similar chemotherapy backbone of taxane followed by anthracycline-containing chemotherapy. The CHER-LOB study of 121 patients demonstrated near doubling of pCRs with dual HER2 targeting (47% vs. 25%, *P* = 0.019) compared with trastuzumab alone [35]. LPT 109096 included 100 patients and demonstrated a significantly higher pCR rate for the combination (74% vs. 54%) compared with trastuzumab alone [39]. A larger randomized phase III trial, NSABP B41, enrolled 529 patients and demonstrated numerically but not statistically improved breast and lymph node pCR for the combination arm versus trastuzumab (60.2% vs. 49.4%, *P* = 0.056) [36].

The TRIO US B07 study randomized 130 patients to treatment with a chemotherapy backbone of docetaxel and carboplatin and trastuzumab, lapatinib or both. The primary endpoint was the pCR rate in the breast and axilla (trastuzumab 47%, lapatinib 25%, combination 52%) [38].

Finally, the nonrandomized window-of-opportunity study TBCRC 006 treated 66 patients with the combination trastuzumab and lapatinib without a chemotherapy backbone for 12 weeks [32]. Women with ER-positive tumors also received letrozole (plus a luteinizing hormone-releasing hormone agonist if premenopausal). Overall, the pCR rate for the breast and axilla was 22% (ER-negative, 28%; ER-positive, 18%). Future studies are needed to identify a subset of HER2-positive breast cancer patients who may not need chemotherapy and can be treated with more complete blockade of HER receptors.

### Pertuzumab and trastuzumab

One proposed cause for resistance to trastuzumab and lapatinib is the overexpression of HER3 in response to tyrosine kinase inhibition [20]. HER3 activation by binding its ligand results in a conformational change of HER3 with the formation of heterodimers with HER2. The formation of HER2-HER3 heterodimers leads to the activation of the PI3K signaling pathway and subsequent increase in cell proliferation and survival. In preclinical models, pertuzumab and trastuzumab combination demonstrated enhanced antitumor activity when compared with each agent alone and in tumors progressing on trastuzumab in HER2-positive breast xenografts [41]. The studies also demonstrated a sustained (>99 days) prevention of metastatic tumor spread to the lungs and liver with the combination in the KPL-4 xenograft model; monotherapy did not prevent these metastases [41].

#### Clinical studies

In the metastatic setting, a phase III trial (CLEOPATRA) of docetaxel and trastuzumab with or without pertuzumab as first-line treatment for 808 patients with HER2-positive MBC showed a significant improvement in progression-free survival and OS with the addition of pertuzumab [18],[42]. A significant prolongation of progression-free survival was observed in the pertuzumab group (HR, 0.62; *P* <0.001) with acceptable toxicity. As a result of this study, in 2012 the US FDA approved the use of pertuzumab in the first-line metastatic setting. The OS was updated in 2013; the median OS was 37.6 months in the placebo group and was not yet reached in the pertuzumab group (HR, 0.66; *P* = 0.0008) [18].

Two neoadjuvant phase II studies have evaluated the addition of pertuzumab to various chemotherapy backbones (Table 3). NeoSphere is a four-arm randomized multicenter, open-label, phase 2 study of neoadjuvant therapy in 417 patients and compares single-agent trastuzumab or pertuzumab with docetaxel or the combination of trastuzumab and pertuzumab with or without docetaxel for four cycles [19]. The patients given pertuzumab and trastuzumab with docetaxel had significant improvement in breast pCR compared with the docetaxel and trastuzumab only arm (46% vs. 29%, *P* = 0.0141) as well as in the breast and lymph node pCR (39.3% vs. 21.5%). Consistent with previous studies, a lower pCR rate was noted for hormone receptor-positive patients compared with hormone receptor-negative tumors (26% vs. 63%). The combination targeted therapies only arm also had a 16.8% breast-only pCR rate and an 11.2% breast and lymph node pCR rate [19].

TRYPHAENA, an open-label, phase II study, randomized 225 patients to neoadjuvant chemotherapy with either: fluorouracil, epirubicin and cyclophosphamide concurrent with combination trastuzumab and pertuzumab for three cycles followed by docetaxel and the combination for three cycles (Arm A); with fluorouracil, epirubicin and cyclophosphamide alone for three cycles followed by docetaxel with the combination for three cycles (Arm B); or with docetaxel, carboplatin and trastuzumab with pertuzumab for six cycles (Arm C) [43]. The pCRs in the breast and lymph nodes were 50.7% (Arm A), 45.3% (Arm B) and 51.9% (Arm C). The study was not intended to evaluate the superiority of any trial arm and the combination of trastuzumab and pertuzumab was generally well tolerated.

In 2013 the US FDA provided an accelerated approval for the use of pertuzumab in combination with trastuzumab and chemotherapy for the neoadjuvant treatment of HER2-positive locally advanced, inflammatory or early-stage breast cancer (either >2 cm in diameter or node-positive) as part of complete treatment regimen for early breast cancer [44]. This approval was based on the results of phase II NeoSphere [19] and was supported by phase II study TRYPHAENA trials [43], added to the fact that there was an already demonstrated improvement in OS in the metastatic setting (based on the CLEOPATRA study [18]). This represented the first regimen approved by the US FDA specifically for neoadjuvant treatment of breast cancer, although full approval will depend on additional data related to EFS in the adjuvant setting, which will be based on the data from the APHINITY trial (expected around 2016 or 2017; NCT01358877).

## Dual blockade with antibody-drug conjugate and targeted therapy

Even with dual blockade, there is a subset of patients that do not achieve pCR or have early progression [19],[42],[43]. In the hope of improving efficacy, studies of ADC-containing dual blockade are ongoing.

### T-DMand pertuzumab

#### Preclinical studies

In MDA-175 cells, the combination of T-DM1 and pertuzumab showed enhanced antiproliferative activity and induction of apoptosis compared with either agent alone [45]. In Calu-3, BT-474 and SK-BR-3 cells, T-DM1 was more active than pertuzumab and the combination was more potent than the single drugs. Previous studies have noted that the presence of the HER3 ligand heregulin (NRG-1β) can reduce the cytotoxic activity of T-DM1 in a subset of breast cancer cell lines. The addition of pertuzumab fully restored the apoptotic response to T-DM1, providing additional evidence for the rationale of combining pertuzumab and T-DM1 [45].

The combination of T-DM1 and pertuzumab *in vivo* in the KPL-4 breast tumor xenografts resulted in statistically significant inhibition of tumor growth as compared with the single-agent treatment group. Sustained tumor growth inhibition was also seen for the duration of the study (88 days) compared with 40 days with T-DM1 alone [45].

#### Clinical studies

A global phase Ib/II study was conducted to investigate the safety and efficacy of T-DM1 and pertuzumab. The phase Ib results demonstrated acceptable tolerability and promising efficacy (response rate 44.4%) in heavily pretreated MBC patients [45]. The MARIANNE trial is a phase III study in first-line HER2-positive MBC patients that randomizes patients to receive trastuzumab plus a taxane versus T-DM1 plus placebo versus T-DM1 plus pertuzumab (results expected late 2014 or early 2015; NCT01120184).

### T-DMand lapatinib

#### Preclinical studies

**Figure 1:**
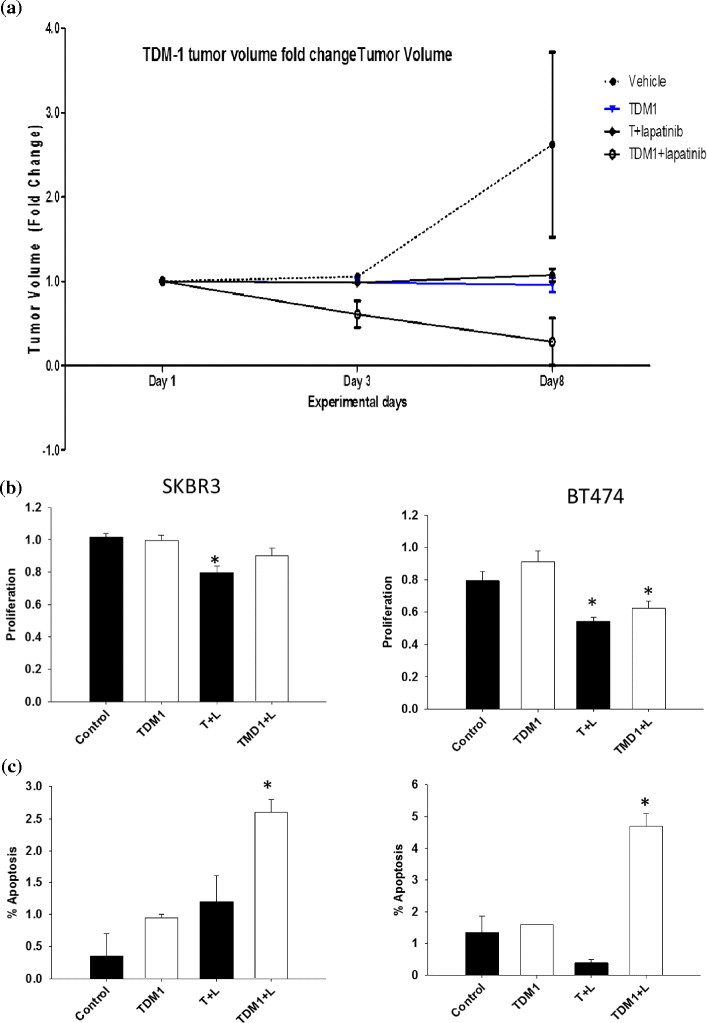
**Dual blockade with antibody-drug conjugate and targeted therapy. (a)** SCID Beige mice were injected with 1 million cells per mouse of the estrogen receptor-positive, human epidermal growth factor receptor (HER)-positive cell line BT474-m1. These animals were randomized into six groups and treated with: vehicle control; trastuzumab (5 mg/kg once weekly); lapatinib (100 mg/kg daily); ado-trastuzumab-emtansine (TDM1; 5 mg/kg weekly); trastuzumab (5 mg/kg once weekly) + lapatinib (100 mg/kg daily); or TDM1 (5 mg/kg weekly) + lapatinib (100 mg/kg daily). Tumor volume fold-change will be measured twice weekly post-treatment. **(b,c)** BT474 and SKBR3 HER2-positive cell lines were treated with the following: vehicle control; TDM1 (1 mg/ml); trastuzumab (10 mg/ml) + lapatinib (10 mM); or TDM1 (1 mg/ml) + lapatinib (10 mM). Cells were assessed for proliferation and apoptosis post-treatment. *Data analyzed by one way analysis of variance followed by Tukey analysis for a pairwise comparison of different groups, *P* < 0.05; T, trastuzumab; L, lapatinib. Data from J Chang, unpublished data.

Single-agent trastuzumab, lapatinib or T-DM1 and the combination of trastuzumab plus lapatinib and the combination of T-DM1 plus lapatinib were studied *in vivo* in the BT474-me breast cancer cell line (J Chang, unpublished data). Tumors treated with single-agent T-DM1 and those treated with the combination of trastuzumab plus lapatinib showed a similar reduction in tumor size. The most significant tumor size reduction was observed in the group treated with the combination of TDM-1 plus lapatinib, where tumor regression was observed in the first 2 days and was significantly superior to the regression observed in the tumors treated with trastuzumab plus lapatinib combination (Figure 1a).

BT474 and SKBR3 cell lines were treated with single-agent trastuzumab, lapatinib, or T-DM1 or with the combination of lapatinib and T-DM1 (J Chang, unpublished data). Proliferation was reduced by lapatinib and lapatinib combination in both cell lines (Figure 1b). Single-agent trastuzumab, single-agent TDM-1 and the combination of TDM-1 plus lapatinib demonstrated significantly induced apoptosis in both cell lines (Figure 1c). These data support the different mechanisms of action of lapatinib versus trastuzumab versus T-DM1, thus indicating the potential for synergism with combinatorial treatments.

#### Clinical studies

Based on the preclinical data, a phase Ib study of lapatinib in combination with T-DM1 and nab-paclitaxel is currently ongoing. In a 3 + 3 study design, the maximum tolerated dose was found to be T-DM1 3.6 mg/kg intravenously every 3 weeks plus lapatinib 750 mg/daily orally along with nab-paclitaxel 80 mg/m^2^ intravenously weekly [ClinicalTrials.gov: NCT02073916].

## Newer tyrosine kinase inhibitors of HER2

Studies are ongoing into the combination of afatinib and trastuzumab or the combination of neratinib and trastuzumab in the metastatic and neoadjuvant settings.

### Trastuzumab and afatinib

Afatinib is a tyrosine kinase inhibitor with activity against HER2 and endothelial growth factor receptor. In a phase II trial of patients with HER2-positive MBC that had progressed following trastuzumab, a 10% response rate (4/41 patients) was observed with afatinib, demonstrating the activity of this agent in this refractory metastatic setting [46].

### Trastuzumab and neratinib

Neratinib is an oral irreversible inhibitor of endothelial growth factor receptor and HER2 tyrosine kinases. A phase II trial of single-agent neratinib showed a 24% response rate in MBC patients who had progression following trastuzumab and a 56% response rate as first-line therapy [47]. Diarrhea occurred in 30% of patients with prior trastuzumab treatment and 13% of patients with no prior trastuzumab treatment [47].

## Different subsets of HER2-positive breast cancers: hormone receptor-positive and hormone receptor-negative subsets

Several completed neoadjuvant trials have demonstrated significant differences in the rates of pCR among hormone receptor-positive and hormone receptor-negative subsets, with higher pCR rates (at least twice as high) in the hormone receptor-negative subsets (Table 4). The highest pCR rates were seen in the HER2-enhanced subset, with the lowest response rates noted in the luminal subsets [37]. The duration of neoadjuvant therapy is relatively short and recent studies have shown pCR correlation with progression-free survival in hormone receptor-negative subsets rather than hormone receptor-positive subsets, so the true benefit of dual therapy in hormone receptor-positive patients should be assessed with longer term therapy. This benefit is currently being assessed with an extension study of lapatinib plus trastuzumab with or without endocrine therapy (HELEX) TBCRC 023 [ClinicalTrials.gov: NCT00999804].

**Table 4:** **Pathologic complete response with dual HER2 regimen in hormone receptor-negative versus hormone receptor-positive subsets of HER2 positive breast cancer: an exploratory comparison**

Study	pCR breast and lymph node combination T + L or T + P (%)	pCR breast and lymph node combination T + L or T + P ER-negative subset (%)	pCR breast and lymph node combination T + L or T + P ER-positive subset (%)
NeoALTTO [12],[34]	46.8	61.3	41.6
CHER-LOB [35]	46.7	41.3	28.8
NSABP 41 [36]	60.2	55.6	73
CALGB 40601 [37]	52	66	42
TRIO US B07 [38]	52	67	40
NeoSphere [19],[32]	39.3	63.2	26
TRYPHAENA [43]	50.7 FECPH → DPH	79.4	46.2
	51.9 TCHP	83.8	50
LPT 109096 [39]	74	NR	NR

DPH, docetaxel, pertuzumab and trastuzumab; ER, estrogen receptor; FECPH, fluorouracil, epirubicin, cyclophosphamide, pertuzumab and trastuzumab; HER, human epidermal growth factor receptor; L, lapatinib; NR, not reported; P, pertuzumab; pCR, pathologic complete response; T, trastuzumab; TCHP, docetaxel, carboplatin, trastuzumab and pertuzumab.

## Conclusion

Therapies directed at HER2 establish a successful treatment paradigm, but *de novo* and acquired resistance exist. With the help of a neoadjuvant model, many studies have demonstrated that single-agent HER2-targeted therapies are efficacious but response is incomplete. Large randomized clinical trials have also demonstrated that dual HER2 targeted combinations with trastuzumab/lapatinib and trastuzumab/pertuzumab are synergistic. Recently, the ADC T-DM1 has been approved for the treatment of HER2-overexpressing MBC. Combination of dual HER2-targeted treatments with T-DM1 and lapatinib or pertuzumab may demonstrate improved efficacy in patients. The results of the ALTTO trial highlight the challenge of determining which patients need multiple targeted therapies. In the future, correlative science embedded within the clinical trials will be invaluable in developing personalized therapy. Studies aimed at characterizing the different genetic alterations associated with resistance to HER2-directed therapies may lead to the discovery of new targets that may overcome resistance.

## Note

This article is part of a series on *`Recent advances in breast cancer treatment*-*,* edited by Jenny Chang. Other articles in this series can be found at http://breast-cancer-research.com/series/treatment.

## Authors’ original submitted files for images

Below are the links to the authors’ original submitted files for images. Authors’ original file for figure 1

## Abbreviations

ADC: Antibody-drug conjugate
DFS: Disease-free survival
EFS: Event-free survival
ER: Estrogen receptor
FDA: Food and Drug Administration
HER: Human epidermal growth factor receptor
HR: Hazard ratio
MBC: Metastatic breast cancer
OS: Overall survival
pCR: Pathologic complete response
PI3K: Phosphoinositide-3-kinase
PTEN: Phosphatase and tensin homolog
T-DM1: Ado-trastuzumab-emtansine
