# Erratum to: Dual HER2 blockade: preclinical and clinical data

**DOI:** 10.1186/s13058-014-0468-9

**Published:** 2014-11-06

**Authors:** Tejal A Patel, Bhuvanesh Dave, Angel A Rodriguez, Jenny C Chang, Edith A Perez, Gerardo Colon-Otero

**Affiliations:** 1 Houston Methodist Cancer Center, 6445 Main Street, P21-34, Houston, 77030 TX USA; 2 Department of Medicine, Weill Cornell Medical College, 1300 York Avenue, New York, 10065 NY USA; 3 Division of Hemotology and Oncology, College of Medicine, Mayo Clinic, 4500 San Pablo Road South, Jacksonville, 32224 FL USA

## Abstract

No abstract.

**Electronic supplementary material:**

The online version of this article (doi:10.1186/s13058-014-0468-9) contains supplementary material, which is available to authorized users.

## Erratum

After publication of our review [1], we noted errors to the legend of Figure 1B, C. The ado-trastuzumab-emtansine concentration should be 1 μg/ml instead of 1 mg/ml. The trastuzumab concentration should be 10 μg/ml instead of 10 mg/ml. The lapatinib concentration should be 10 μM instead of 10 mM (Please see Figure 1, a corrected version of the original Figure 1).

**Figure 1:**
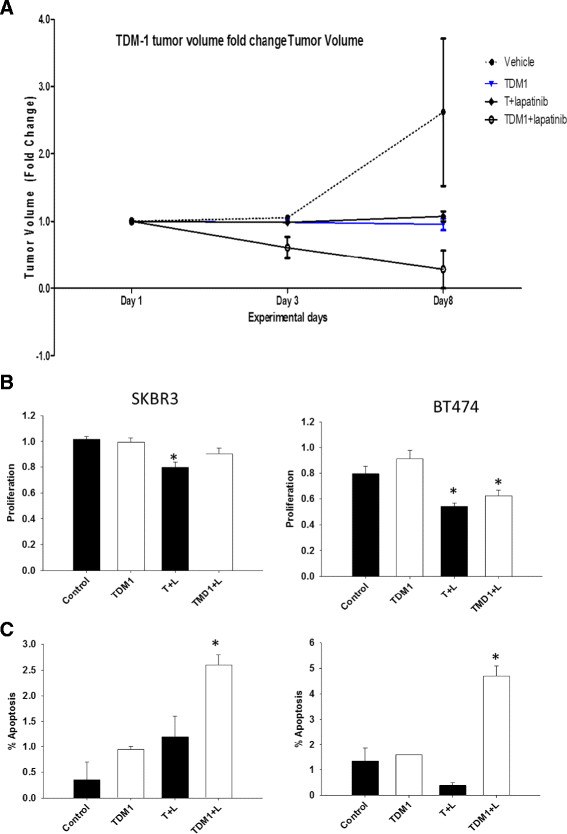
**Dual blockade with antibody–drug conjugate and targeted therapy. (A)** SCID Beige mice were injected with 1 million cells per mouse in the estrogen receptor-positive, human epidermal growth factor receptor (HER)-positive cell line called BT474-m1. These animals were randomized into six groups and treated with: vehicle control; trastuzumab (5 mg/kg once weekly); lapatinib (100 mg/kg daily); ado-trastuzumab-emtansine (TDM-1; 5 mg/kg weekly); trastuzumab (5 mg/kg once weekly) + lapatinib (100 mg/kg daily); or TDM-1 (5 mg/kg weekly) + lapatinib (100 mg/kg daily). Tumor volume fold-change will be measured twice weekly post treatment. **(B)**, **(C)** BT474 and SKBR3 HER2-positive cell lines were treated with the following: vehicle control; TDM-1 (1 μg/ml); trastuzumab (10 μg/ml) + lapatinib (10 μM) 4); or TDM-1 (1 μg/ml) + lapatinib (10 μM). Cells were assessed for proliferation and apoptosis post treatment. *Data analyzed by one way analysis of variance followed by Tukey analysis for a pairwise comparison of different groups, *P* < 0.05. T, trastuzumab; L, lapatinib. Data from J Chang, unpublished data.

## Authors’ original submitted files for images

Below are the links to the authors’ original submitted files for images. Authors’ original file for figure 1
