# Supplementary figures and images for: Dual HER2 blockade: preclinical and clinical data
Source: Breast Cancer Res. 2014 Jul 31;16:419. doi: 10.1186/s13058-014-0419-5 (PMC4429364; doi:10.1186/s13058-014-0419-5)

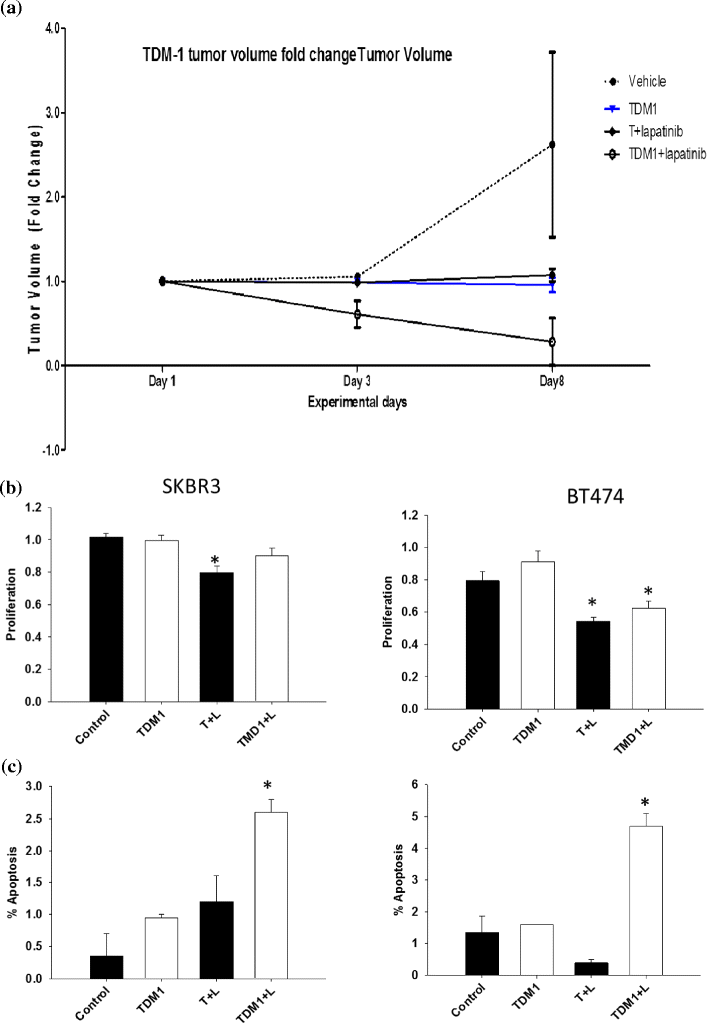

Supplement: Supplementary file 1 — Authors’ original file for figure 1 [file 13058_2014_419_MOESM1_ESM.gif]
