# Supplementary figures and images for: Erratum to: Dual HER2 blockade: preclinical and clinical data
Source: Breast Cancer Res. 2014 Nov 6;16:468. doi: 10.1186/s13058-014-0468-9 (PMC4303199; doi:10.1186/s13058-014-0468-9)

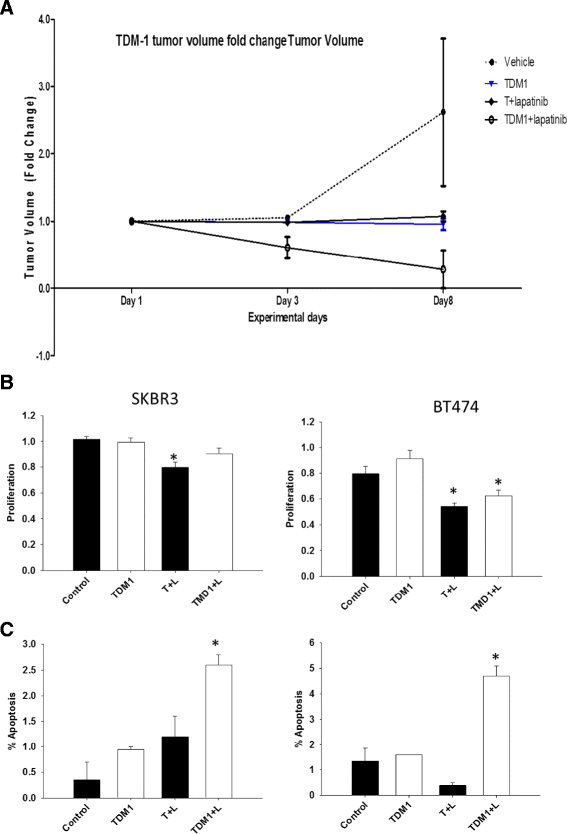

Supplement: Supplementary file 1 — Authors’ original file for figure 1 [file 13058_2014_468_MOESM1_ESM.gif]
